# Association of *ADRB2* gene polymorphisms and intestinal microbiota in Chinese Han adolescents

**DOI:** 10.1515/biol-2022-0646

**Published:** 2023-08-01

**Authors:** Shanrong Xu, Wenqi Liu, Li Gong, Xinyang Li, Wenwen Chu, Meng Han, Shuiqin Shi, Duoqi Zhou

**Affiliations:** College of Life Science, Anqing Normal University, Anqing, 246133, P. R. China; Department of Clinical Laboratory, Chongqing General Hospital, Chongqing, 400014, P. R. China; Chongqing Traditional Chinese Medicine Hospital, Chongqing, 400021, P. R. China; College of Life Science, Anqing Normal University, 1318 North Jixian Road, Anqing, 246133, P. R. China

**Keywords:** Chinese Han adolescents, *ADRB2* gene polymorphisms, intestinal microbiota, correlation analysis

## Abstract

Gut microbiota are closely related to health, and the β2-adrenergic receptor (*ADRB2*) gene is associated with gastrointestinal diseases. However, little is known about the relationship between *ADRB2* gene polymorphisms and intestinal microbiota. In the present study, we aimed to explore the relationship between *ADRB2* gene polymorphisms and gut microbiota in Chinese Han adolescents. Data analysis showed that the relative abundance, PICRUSt function prediction, and Chao1 and ACE indices of gut microbiota were significantly different between males and females (*P* < 0.05). The rs1042711 was positively associated with the relative abundance of Actinobacteria, Coriobacteriia, Bifidobacteriales, Erysipelotrichi, and Erysipelotrichales. The rs12654778 was negatively associated with Bacilli, Lactobacillales, Bacteroidaceae, and *Bacteroides*. rs1042713 was positively associated with Lactobacillales and Bifidobacteriales. The rs1042717 was positively associated with Bifidobacteriales and negatively associated with Veillonellaceae. The rs1042719 was negatively associated with Erysipelotrichi and Erysipelotrichales and positively associated with Erysipelotrichi, Erysipelotrichales, Bifidobacteriales, and Ruminococcaceae in females. The rs1801704 was positively associated with Erysipelotrichi, Erysipelotrichales, Bifidobacteriales, Actinobacteria, Coriobacteriia, and Bifidobacteriales. The rs2053044 was positively associated with Ruminococcaceae, *Dialister*, Firmicutes, Clostridia, Clostridiales, Bifidobacteriales, and *Faecalibacterium* and negatively associated with Bacilli, Lactobacillales, Lachnospiraceae, and Porphyromonadaceae (*P* < 0.05). These results suggested that the relative abundance, diversity, and PICRUSt function predictions of male and female gut microbiomes differ significantly and that *ADRB2* gene polymorphisms were associated with gut microbiome abundance in Chinese Han adolescents.

## Graphical abstract

Gut microbiota are closely related to health, and the β2-adrenergic receptor gene (*ADRB2*) is associated with gastrointestinal diseases. In the present study, we aimed to explore the relationship between *ADRB2* gene polymorphisms and gut microbiota in Chinese Han adolescents. The relative abundance, diversity, and PICRUSt function predictions of male and female gut microbiomes differ significantly and that *ADRB2* gene polymorphisms were associated with gut microbiome abundance in Chinese Han adolescents.

**Figure:**
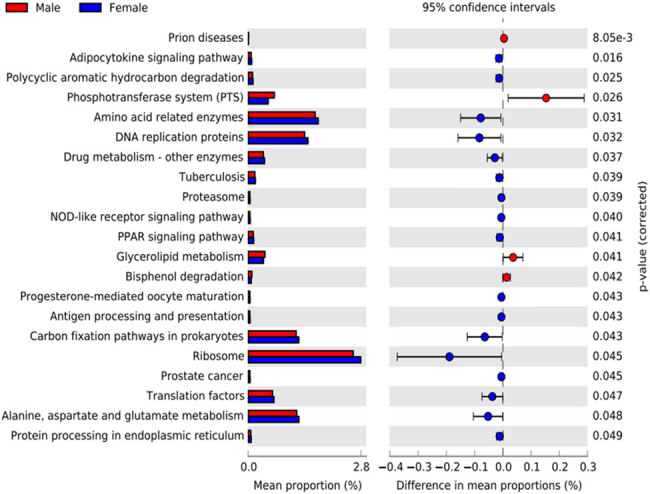

## 1 Introduction

The human gut is a vast reservoir of microorganisms, including bacteria, fungi, viruses, archaea, and protozoa. Over the past decade, the microbiome has been recognized as an important factor in human health, playing a role in nutrient absorption, metabolism, and resistance to harmful bacteria and exerting immunomodulatory, anti-aging, anti-tumor, and other effects [1,2]. Imbalances of gut microbiota are associated with obesity, diabetes, autism, and gastrointestinal diseases such as colorectal cancer [3,4,5]. Xu et al. found that in the gut of ulcerative colitis patients, the abundance of *Roseburia intestinalis* was significantly reduced [6]. Ibrahim et al. found that colitis-induced colorectal cancer and *ESR1* affect the diversity of gut microbiota [7], but the molecular mechanisms by which gut microbiota affect human health are poorly understood. Therefore, many studies focus on the factors affecting the composition and structure of the gut microbiota. Studies have found not only that geographics, drugs, living habits, and exercise changed the composition of the intestinal flora [8,9,10,11] but also that sex and genetics were important factors affecting gut microbiota [12,13].

With the development of large-scale genotyping methods and multiple sequencing technologies, Crespo-Piazuelo et al. performed genome-wide association studies of pig genotypes and their gut microbiota composition and found that 52 single-nucleotide polymorphisms (SNPs) codistributed in 17 regions along the pig genome were associated with the relative abundance of six genera, indicating an association between the host genome and gut microbiota in pigs [14]. The relationship between the abundance of *Bifidobacterium* and SNPs near the lactase gene was also investigated in humans [15]. β2-Adrenergic receptor (*ADRB2*) is localized on chromosome 5q31–q32 and has a length of 1.8 kb. It is an intron-free gene encoding 413 amino acids [16]. It is widely expressed in the gastrointestinal tract [17]. A growing body of evidence has shown that the promoter region of *ADRB2* contains several SNPs, including rs1801704 (−20T/C), rs1042711 (−47T/C), rs11959427 (−367T/C), rs11168070 (−468C/G), rs12654778 (−654G/A), rs2053044 (−1023G/A), rs2400707 (−1343A/G), and rs2895795 (−1429T/A), some of which are present in putative regulatory elements and could influence gene expression [18]. *ADRB2* has been found to play an important role in the progression of gastrointestinal diseases [19,20]. Zhi et al. found that autophagy played an active role in the development of chronic stress-induced gastric cancer and that the *ADRB2* gene negatively regulated the autophagy signaling pathway [21]. Pan et al. found that SNPs in *ADRB2* increased the risk of pancreatic cancer [22]. It is unknown, however, whether *ADRB2* polymorphisms are associated with gut microbiota.

We hypothesized that SNPs in the promoter region of the *ADRB2* gene affect the abundance of the intestinal flora. To address this hypothesis, we analyzed whether the intestinal microflora abundance of adolescents in China is related to *ADRB2*. A comprehensive assessment was conducted to determine the SNPs and to study the association between *ADRB2* and the abundance of intestinal flora in adolescents in China. Our study provides insight into the effects of *ADRB2* polymorphisms.

## 2 Materials and methods

### 2.1 Volunteer recruitment

In the present study, 91 healthy Chinese Han college students aged 19−25 years (21.60 ± 1.38) were recruited (50 females and 41 males). Participants suffering from any symptoms of constipation, bloody stool, diarrhea, or any other gastrointestinal disease and participants who had taken antibiotics in the past 3 months were excluded. Baseline characteristics of the participants are available in Table 1.

**Table 1:** Baseline characteristics of the participants

Sex	Number	Age (years)	Age range	Height (cm)	Weight (kg)
Total	91	21.60 ± 1.38	19–25	167.49 ± 9.30	66.64 ± 16.17
Male	41	21.34 ± 1.28	19–24	175.34 ± 6.33	78.33 ± 14.46
Female	50	21.82 ± 1.44	19–25	161.06 ± 5.71	57.06 ± 10.07


**Informed consent:** Informed consent has been obtained from all individuals included in this study.
**Ethical approval:** The research related to human use has been complied with all the relevant national regulations, institutional policies and in accordance with the tenets of the Helsinki Declaration, and has been approved by Ethics Committee of Anqing Normal University (AQNU2018018H).

### 2.2 Sample collection

Participants were provided with a home stool collection kit to collect one stool sample, which was stored at room temperature. Moreover, 5 mL of venous blood was collected with a disposable vacuum blood collection tube and plasma was stored at −80℃. All samples were collected within 24 h after filling in the questionnaire. For 10 h before blood collection, participants fasted, did not drink alcohol, and did not stay up late. Sampling was performed using standard protocols.

### 2.3 Blood DNA extraction and gene sequencing

A Tiangen DNA extraction kit (Tiangen, China) was used to extract DNA from the blood samples. A NanoDrop ND-1000 spectrophotometer (Thermo Fisher Scientific, Waltham, MA, USA) was used to determine the concentration and purity of DNA, and the integrity and size of DNA were analyzed by 1% agarose gel electrophoresis. Next, the DNA samples were amplified by MSA6 plates. To obtain the SNPs of the target gene, the gene polymorphism sites were sequenced using a high-throughput Illumina NovaSeq6000 sequencing platform with an Illumina CGA gene chip.

### 2.4 Fecal DNA extraction and 16S ribosomal RNA gene sequencing

The PowerMax (stool/soil) DNA isolation kit (MoBio Laboratories, Carlsbad, CA, USA) was used to extract the total fecal DNA, which was stored at −20℃ until further analysis. The quantity and quality of extracted DNA were measured using a NanoDrop ND-1000 spectrophotometer (Thermo Fisher Scientific, Waltham, MA, USA) and by agarose gel electrophoresis, respectively. High-throughput sequencing of the bacterial 16S rRNA gene was conducted by Hangzhou Guhe Biotechnology Co., Ltd. (Hangzhou, China). The V4 region of the bacterial 16S rRNA marker gene was PCR amplified using the primers 515F (5′-GTGCCAGCMGCCGCGGTAA-3′) and 806R (5′-GGACTACHVGG GTWTCTAAT-3′) as previously reported. The amplicons were purified with Agencourt AMPure XP Beads (Beckman Coulter, Indianapolis, IN, USA) and quantified using the PicoGreen dsDNA Assay Kit (Invitrogen, Carlsbad, CA, USA). Purified amplicons were used in equimolar amounts for paired-end 2 × 150 bp sequencing on an Illumina MiSeq6000 platform by GUHE Info Technology Co., Ltd. (Hangzhou, China).

### 2.5 Sequencing data analysis

The 16S rRNA sequence data analyses were mainly performed using QIIME (version 1.9.1) and R packages (v3.2.0). Paired-end reads were assembled and operational taxonomic units (OTUs) were clustered with a 97% similarity cut-off using Vsearch V2.4.4 (--fastq_mergepairs --fastq_minovlen 5). A representative sequence was selected from each OTU using default parameters. OTU taxonomic classification was conducted by VSEARCH by searching for the representative sequences in the Greengenes database. OTU-level alpha-diversity indices such as the Chao1 richness estimator were calculated using the OTU table in QIIME. KEGG pathway enrichment analysis was conducted using Stamp (version 2.1.3). The linear discriminant analysis effect size (LEfSe) was used to assess the differences in relative abundance between different taxa.

PLINK (V1.07) software was used to screen and perform quality control of the acquired SNP data. The following threshold values were used: minor allele frequency (MAF) >0.05, SNP detection rate >90%, sample detection outcome rate >90%, and Hardy–Weinberg (H–W) balance *P*-value >0.05. After screening, Haploview software (4.1) was used to detect the linkage equilibrium of genes.

H–W equilibrium, genotype frequencies, and allele frequencies were analyzed by the chi-square test. Grouped values are reported as the mean, standard error of the mean, and 95% confidence intervals. Between-group comparisons were performed using Student’s *t*-test for continuous variables. A linear regression model and an additive effects model were used, with sex, height, body weight, and age as covariates to correct for the effects of these factors on gut microbiota phenotypic indicators to analyze the association between the genotype of *ADRB2* SNPs and different phenotypes. Statistical analysis was carried out using SPSS Statistics v.20 (IBM, Armonk, NY, USA). Differences were considered significant when *P* < 0.05.

## 3 Results

### 3.1 *ADRB2* SNP information

According to the annotation of sequencing results, the *ADRB2* SNP information Illumina gene chip was used to scan for 12 SNP loci on *ADRB2*. PLINK software was used to control and screen the SNP loci. We found that the rs2053044, rs11959427, rs1801704, rs1042711 (Arg19Cys), rs1042713 (Arg16Gly), rs12654778, rs1042717 (Leu84Leu), rs1042718 (Arg175Arg), and rs1042719 (Gly351Gly) loci met the criteria, so these SNPs were subjected to subsequent association studies. Table 2 provides the basic information for these nine SNP loci, including location, MAF value, genotype count, and H–W balance *P*-value. Next, using Haploview (4.1) we found that rs11959427, rs1042711, rs1801704, rs2053044, and rs1042719 loci were in linkage equilibrium, and rs1801704 was selected as the tag SNP for analysis; rs1042713 and rs12654778 were also in linkage equilibrium, and rs12654778 was selected as the tag SNP; rs1042717 and rs1042718 were also in linkage equilibrium, and rs1042717 was selected as the tag SNP.

**Table 2:** Information of the *ADRB2* gene SNPs included after quality control

Chromosome	SNP	Position	Second allele	Major allele	MAF	Genotype number	H–W *P*-value
5	rs2053044	148205372	A	G	0.2802	7/37/47	1.00
5	rs12654778	148205741	A	G	0.3846	14/42/35	0.83
5	rs11959427	148206028	C	T	0.0714	0/13/78	1.00
5	rs1042711	148206348	C	T	0.0549	0/10/81	1.00
5	rs1801704	148206375	C	T	0.0549	0/10/81	1.00
5	rs1042713	148206440	G	A	0.3956	15/42/34	0.83
5	rs1042717	148206646	A	G	0.3297	13/34/44	0.16
5	rs1042718	148206917	A	C	0.3242	13/33/45	0.10
5	rs1042719	148207447	C	G	0.4890	22/45/24	1.00

### 3.2 Analysis of intestinal flora composition

**Figure 1:**
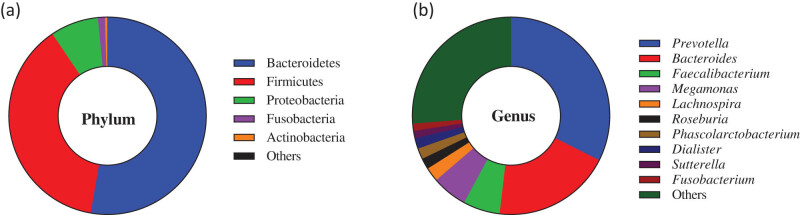
Species composition analysis of participates. Note: (a) The composition on phylum level. (b) The composition on genus level.

A total of 12,199,153 16S rRNA reads were generated. After quality control, 11,539,874 valid sequences were obtained. At the phylum level, the most abundant phylum was Bacteroidete*s* (relative abundance 52.75%), followed by Firmicutes (relative abundance 37.82%), Proteobacteria (relative abundance 7.86%), Fusobacteria (relative abundance 1.17%), and Actinobacteria (relative abundance 0.38%) (Figure 1a). At the genus level, the microbiome mainly contained *Prevotella* (relative abundance 32.44%), *Bacteroides* (relative abundance 19.42%), *Faecalibacterium* (relative abundance 6.05%), *Megamonas* (relative abundance 5.8%), *Lachnospira* (relative abundance 2.31%), *Roseburia* (relative abundance 1.81%), *Phascolarctobacterium* (relative abundance 1.78%), *Dialister* (relative abundance 1.73%), *Sutterella* (relative abundance 1.22%), and *Fusobacterium* (relative abundance 1.17%) (Figure 1b).

**Figure 2:**
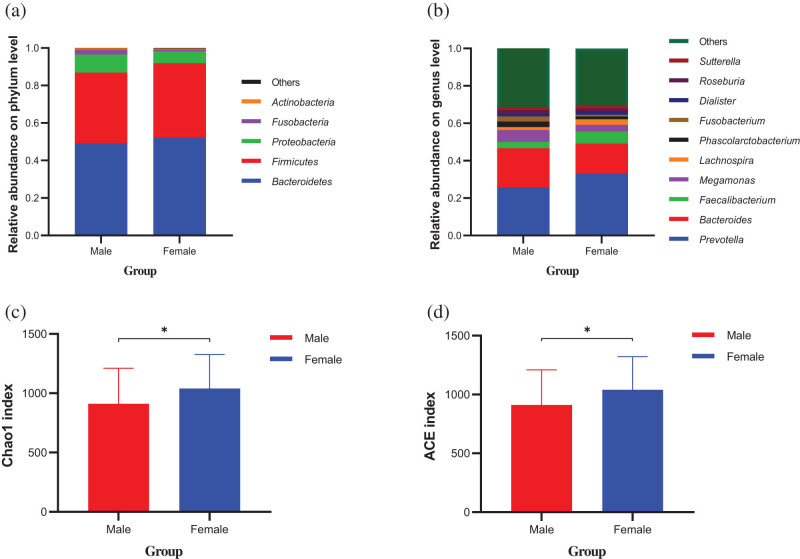
The relative abundance and diversity of gut microbiota in male and female. Note: (a) The relative abundances on phylum level. (b) The relative abundances on genus level. (c) Chao1 index (*P < 0.05). (d) ACE index (*P < 0.05). Red and blue dots indicate the bacterial taxa enriched in male and female subjects, respectively.

**Figure 3:**
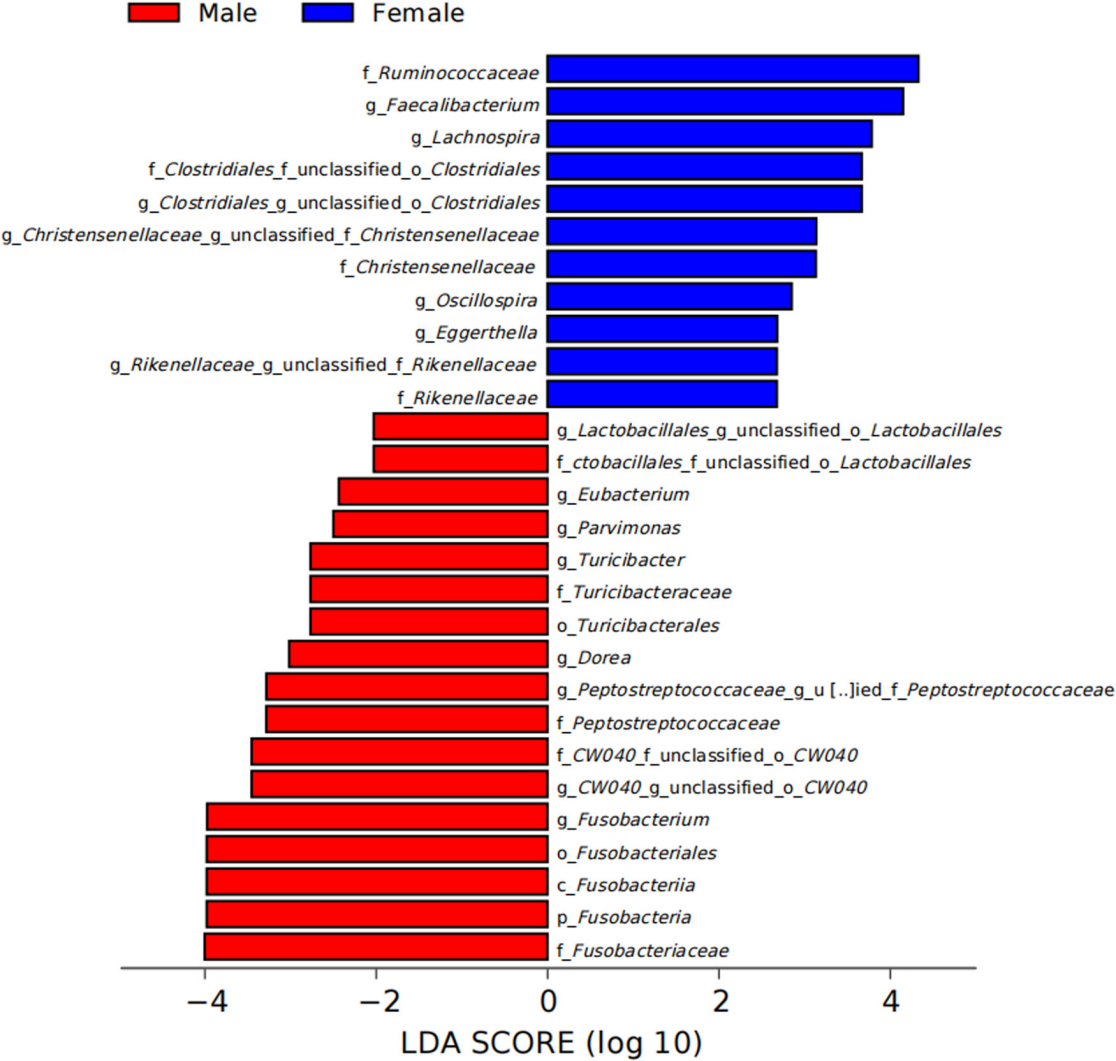
Differentially abundant bacterial taxa in male and female according to a linear discriminant analysis. Note: Red and blue dots indicate the bacterial taxa enriched in male and female subjects, respectively. Only the taxa having an LDA of  >2.0 are shown in the figure.

**Figure 4:**
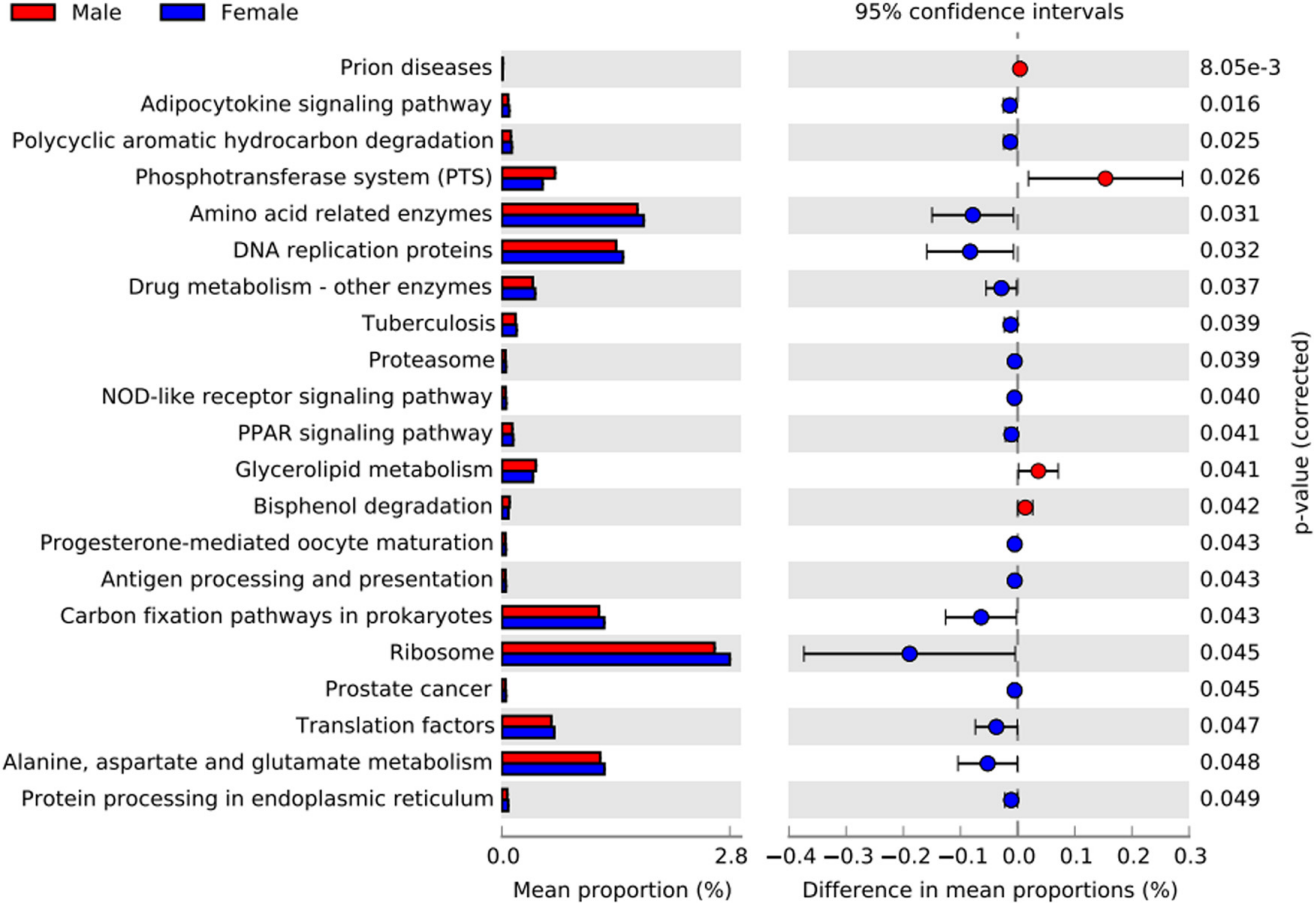
KEGG metabolic function prediction in male and female. Note: Red and blue dots indicate the bacterial taxa enriched in male and female subjects, respectively. Only the taxa having a *P* of  <0.05 are shown in the figure.

To investigate the impact of sex on the gut microbiome, we analyzed the composition, diversity, and KEGG metabolic pathways of the gut microbiota between males and females. At the phylum level, both male and female gut microbiota were mainly composed of Bacteroidetes, Firmicutes, Proteobacteria, Fusobacteria, and Actinobacteria, and at the genus level, they were mainly composed of *Prevotella*, *Bacteroides*, *Faecalibacterium*, *Lachnospira*, and *Megamonas*; however, the relative abundances of gut microbiota were different between the two groups (Figure 2a and b). In addition, we found that the Chao1 and ACE indices also differed between males and females; the community richness was significantly higher in females than in males (Figure 2c and d). Based on the LEfSe data, the Ruminococcaceae, *Faecalibacterium*, *Lachnospira*, Christensenellaceae, *Oscillospira*, *Eggerthella*, and Rikenellaceae were identified as the bacterial taxa enriched in females, while *Eubacterium*, *Parvimonas*, *Turicibacter*, *Dorea*, Peptostreptococcaceae, and *Fusobacterium* were significantly enriched in males (Figures 3 and 4). PICRUSt function prediction in the two groups showed that the relative abundance of 21 functional annotations was significantly different; males were enriched in prions, the phosphotransferase system, glycerolipid metabolism, and bisphenol degradation, and females were enriched in the adipocytokine signaling pathway, polycyclic aromatic hydrocarbon degradation, amino acid-related enzymes, DNA replication proteins, drug metabolism, tuberculosis, the proteasome, the NOD-like receptor signaling pathway, the PPAR signaling pathway, progesterone-mediated oocyte maturation, antigen processing and presentation, carbon fixation pathways in prokaryotes, the ribosome, prostate cancer, translation factors, protein processing in the endoplasmic reticulum, and alanine, aspartate, and glutamate metabolism (Figure 4).

### 3.3 Correlation analysis between *ADRB2* SNPs and intestinal microorganisms

**Figure 5:**
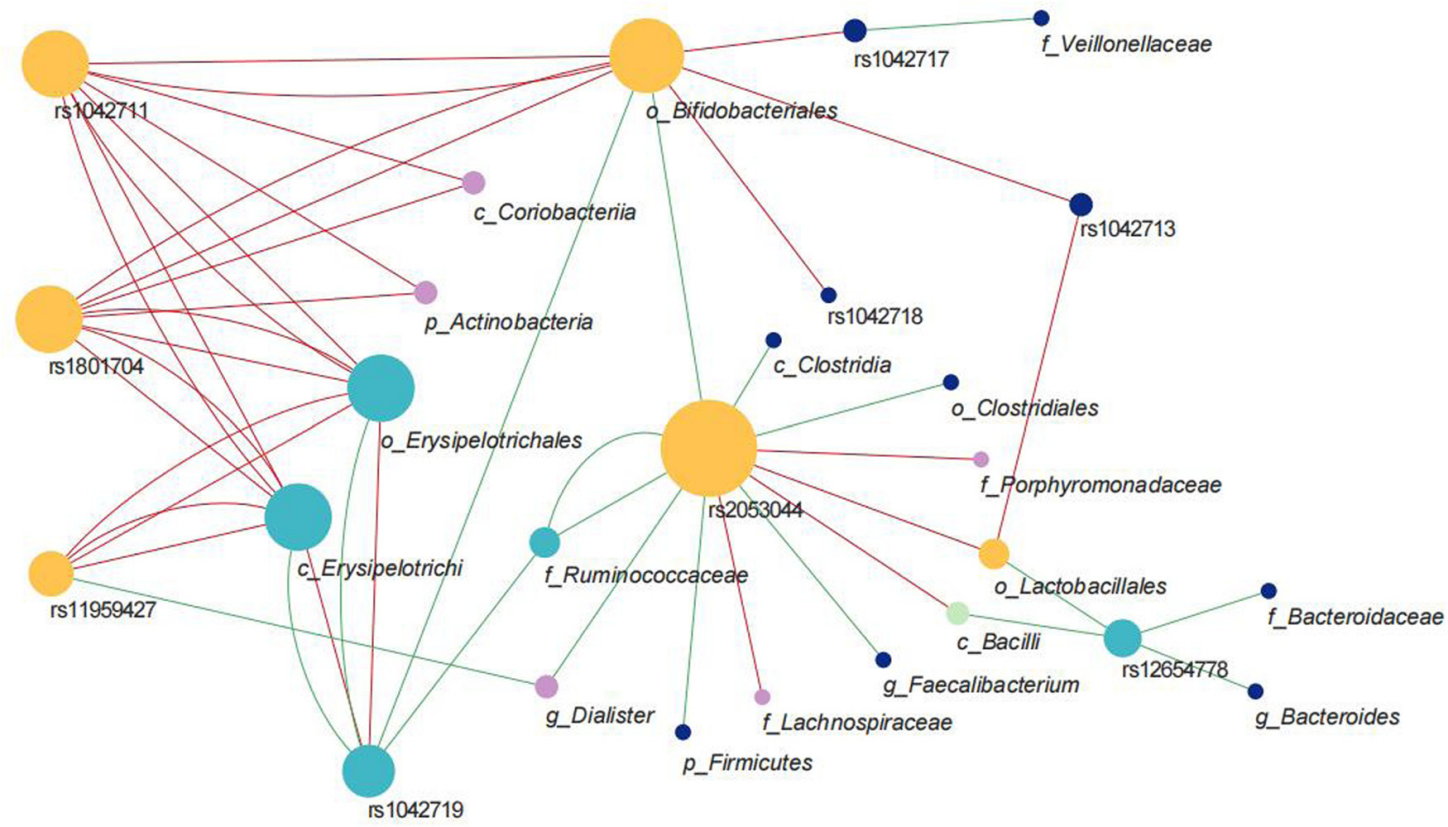
Interaction network between *ADRB2* polymorphism and gut microbiota abundance in total, male, and female. Note: The size of the node is proportional to the degree value, the red line represents the site that has the positive regulation of the flora, and the green line represents the negative regulation.

The interaction network between *ADRB2* SNPs and gut microbiota was generated. The results showed that rs1042711, rs1801704, rs11959427, and rs2053044 were common in the total group, in males, and in females. rs1042711 and rs1801704 had positive effects; the C allele increased the abundance of Erysipelotrichi, Erysipelotrichales, and Bifidobacteriales by 8,711, 8,711, and 5,966 units, respectively, increased the abundance of Actinobacteria, Coriobacteriia, and Bifidobacteriales in males by 1.48 × 10^+4^, 3,641, and 1.23 × 10^+4^ units, respectively, and increased the abundance of Erysipelotrichi and Erysipelotrichales in females by 1.45 × 10^+4^ units. The rs11959427 had negative effects on *Dialister*; the C allele decreased *Dialister* abundance by 2.28 × 10^+4^ units. rs2053044 had the strongest effects; the A allele increased the abundance of Porphyromonadaceae, Lactobacillales, Bacilli, and Lachnospiraceae and decreased the abundance of Bifidobacteriales, Clostridiales, Clostridia, *Faecalibacterium*, Firmicutes, *Dialister*, and Ruminococcaceae (Figure 5). These data are detailed in Tables S1–S3. rs1042719 and rs12654778 were common in the total group and in females. The A allele of rs1042719 decreased the abundance of Erysipelotrichi and Erysipelotrichales in the total group, but in females, the same allele increased the abundance of Erysipelotrichi and Erysipelotrichales. In addition, it decreased the abundance of Ruminococcaceae and Bifidobacteriales in females. The A allele of rs12654778 decreased the abundance of Bacilli in the total group, Lactobacillales in the total group, Bacteroidaceae in females, and *Bacteroides* in females by 7,311, 6,137, 6.24 × 10^+4^, and 6.24 × 10^+4^ units, respectively. Moreover, we found that rs1042713, rs1042717, and rs1042718 were female-specific loci. The G allele of rs1042713 and the A allele of rs1042718 had positive effects on the abundance of functional flora. The A allele of rs1042717 increased the abundance of Bifidobacteriales by 1,772 units and decreased the abundance of Veillonellaceae by 2.44 × 10^+4^ units (Figure 5). These data are detailed in Tables S1 and S3.

**Figure 6:**
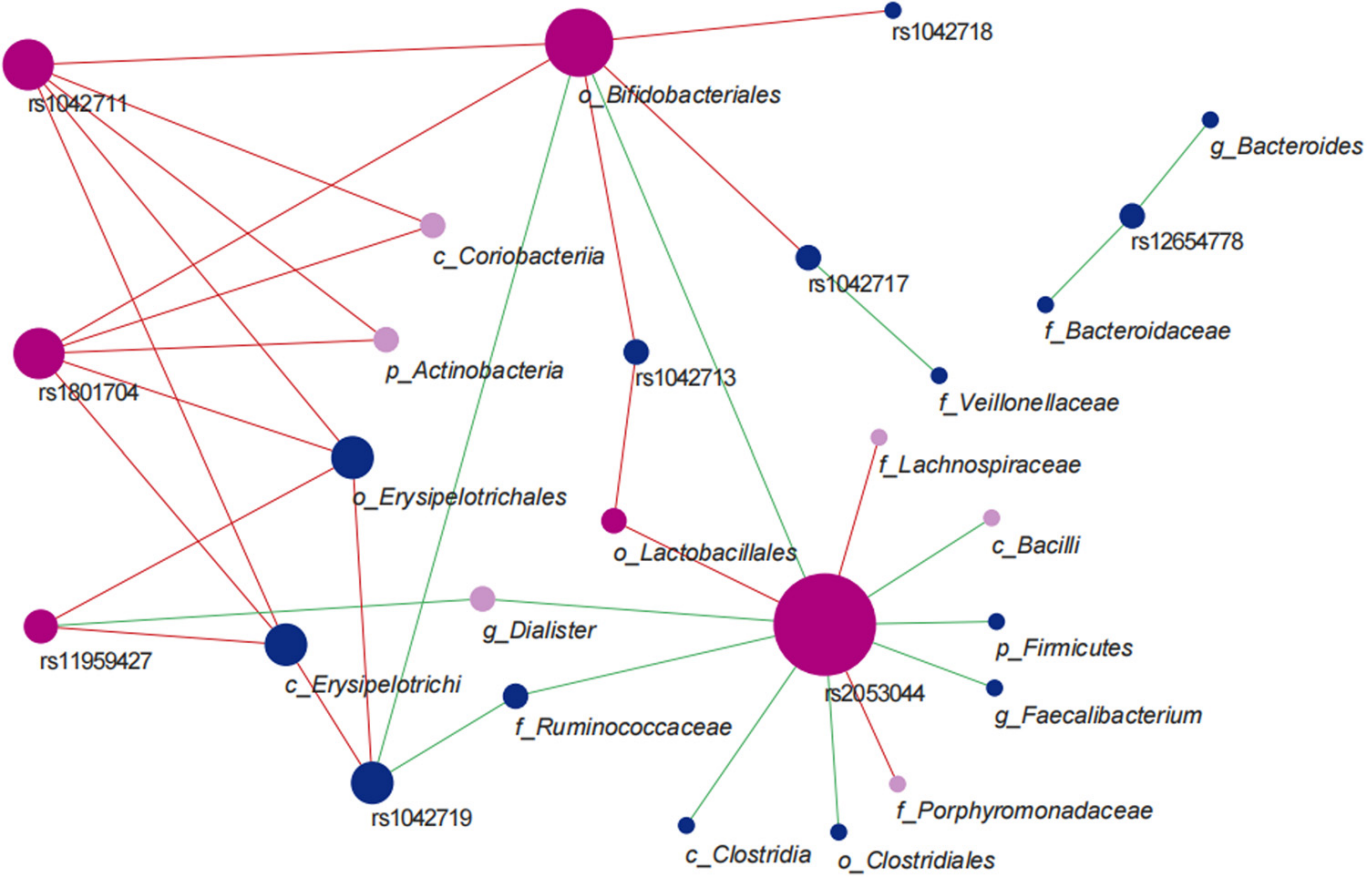
Interaction network between *ADRB2* polymorphism and gut microbiota abundance in male and female. Note: The size of the node is proportional to the degree value, the red line represents the site that has the positive regulation of the flora, and the green line represents the negative regulation.

In males and females, rs1042711, rs1801704, rs2053044, and rs11959427 were common loci. The C alleles of rs1042711 and rs1801704 increased the abundance of functional flora. The C allele of rs11959427 increased the abundance of the female-specific bacteria Erysipelotrichi (by 9,658 units) and Erysipelotrichales (by 9,658 units) and decreased the abundance of the male-specific *Dialister* by 2.28 × 10^+04^ units. The A allele of rs2053044 decreased the abundance of most of its related flora but increased the abundance of Lactobacillales, which is common between males and females, and the male-specific Lachnospiraceae and Porphyromonadaceae. We did not find male-specific loci, but five female-specific loci, among which rs1042713 and rs1042718 played a positive role. The G allele of rs1042713 increased the abundance of Lactobacillales and Bifidobacteriales by 8,784 and 1,998 units, respectively, and the A allele of rs1042718 increased the abundance of Bifidobacteriales by 1,624 units. The A allele of rs12654778 played a negative role; it decreased the abundance of Bacteroidaceae and *Bacteroides* by 6.24 × 10^+04^ units. The G allele of rs1042719 decreased the abundance of Bifidobacteriales and Ruminococcaceae and increased the abundance of Erysipelotrichi and Erysipelotrichales. The rs1042717 increased the abundance of *Bifidobacteriales* and decreased the abundance of Veillonellaceae (Figure 6). These data are detailed in Tables S2 and S3.

## 4 Discussion

The human gut microbiome is acquired at birth. Throughout life, it is involved in host metabolism, immunity, and health. With the rapid development of medicine and life sciences, fecal microbiota transplantation has been applied in the treatment of disease [23]. However, gut microbiota can be influenced by multiple factors, and due to its complex composition and functions [24], there is still a lack of understanding of the relationship between gut microbiota and health and the underlying molecular mechanism. Our experimental results showed that the gut microbiota of Chinese Han students was mainly composed of Bacteroidetes and Firmicutes, which is consistent with previous studies [2]. The gut microbiome composition and diversity of males and females were significantly different, in accordance with a report by Sinha et al., which showed that alpha diversity was higher in females than in males in a Dutch cohort [25]. De la Cuesta-Zuluaga et al. reached the same conclusion in cohorts from the United States, United Kingdom, and Colombia, but they did not find a sex difference in alpha diversity in a Chinese cohort. In addition, they found a higher relative abundance of *Faecalibacterium* and Ruminococcaceae and a lower relative abundance of *Bacteroides* in females. The higher relative abundance of *Prevotella* and lower relative abundance of *Bacteroides* in males contradicted our results [26]. These discrepancies may be related to age, race, and geographic regions. Because several metabolic syndromes and gastrointestinal diseases showed a sex difference and the gut microbiome is involved in various processes, our study may serve as a reference for disease prevention and drug resistance studies.

In addition, according to our present results, *ADRB2* SNPs were related to the abundance of gut microbiota. Among them, the SNP showing the strongest effects was rs2053044, and the taxon showing the most significant differences was Bifidobacteriales. Bifidobacteriales is a family of Gram-positive, anaerobic, branched rod-shaped bacteria with the ability to produce short-chain fatty acids (SCFAs) and lactate as well as specific immune stimuli and to acidify the intestinal environment to protect against disease in early life. SCFAs are absorbed by colonocytes and peripheral tissue and serve as a source of energy or can be used as substrates for lipogenesis, gluconeogenesis, or cholesterol synthesis in the liver. Interestingly, *ADRB2* is involved in the negative regulation of fatty acid synthesis. Our experimental results showed that the *ADRB2* SNP rs2053044 was inversely correlated with the abundance of Bifidobacteriales, which suggested that *ADRB2* may participate in the regulation of Bifidobacteriales through the cAMP signaling pathway. Moreover, we found that rs1801704, rs1042711, rs1042719, and rs1801704 were positively associated with the abundance of Erysipelotrichi and Erysipelotrichales. A high abundance of Erysipelotrichi in the human gut is closely related to the occurrence of fatty liver disease. It has been shown that the interaction of Erysipelotrichi with host enzymatic activity transforms choline into the toxic methylamine, and these transformations reduce the bioutilization of choline, thus promoting fatty liver development. Because adrenergic and cholinergic receptors, both of which are G protein-coupled receptors, have similar mechanisms of action and affect each other, altered *ADRB2* function can affect signal transduction and the function of cholinergic receptors. All these observations suggest a close relationship between *ADRB2* and Erysipelotrichi, and *ADRB2* may play an important role in the development of fatty liver.

There are also some studies that suggest that enrichment of Erysipelotrichi and increased Firmicutes abundance are important features of the gut microbiota in obesity [27,28,29], while the relative abundance of *Lactobacillus* was inversely associated with obesity[30]. Our experimental results showed that rs2053044 was negatively correlated with Firmicutes and positively with *Lactobacillus*. This is inconsistent with the prior conclusion that the rs1042713 A allele is a risk factor for obesity. Actually, some studies have shown that obesity was not related to Firmicutes [31,32]. Therefore, more studies are needed to confirm the relationship between *ADRB2* polymorphisms and obesity-related intestinal flora.

Our experimental results indicated that rs2053044 was also positively correlated with the abundance of Lachnospiraceae and Prophyromonadaceae and negatively associated with Ruminococcaceae, *Dialister*, Clostridia, and *Faecalibacterium*. The rs1042717 was negatively correlated with Veillonellaceae. The rs12654778 was negatively associated with Bacteroidaceae and *Bacteroides*. Previous studies have shown that the abundance of Ruminococcaceae and Lachnospiraceae was decreased in autism spectrum disorder [33]. Barandouzi et al. found that the abundance of Porphyromonadaceae was decreased, while the abundance of Bacteroidaceae and Veillonellaceae was increased in people with depression [34]. The relative abundance of Coriobacteriia was lower in patients with type 1 narcolepsy [35]. The abundance of Clostridia was positively associated with the levels of bile acid excretion in diarrhea-predominant irritable bowel syndrome [36]. *Faecalibacterium* is one of the most important bacteria in the human gut microbiota, accounting for 5–15% of the total number of bacteria detected in healthy human fecal samples, is one of the most important producers of butyric acid, has anti-inflammatory effects, maintains the activity of bacterial enzymes, and protects the digestive system from intestinal pathogens [37]. A decrease in the abundance of this microorganism has been reported in individuals with chronic constipation, celiac disease, irritable bowel syndrome, and inflammatory bowel disease [38,39,40]. *Dialister* is a pathogen that is highly abundant in circulating plasma in patients with cirrhosis [41]. In addition, its increase in abundance is associated with weight gain, and there are also studies showing that *Actinobacteria* and *Dialister* are more abundant in patients with spinal arthritis than in controls [42]. The human gut microbiota is closely related to many diseases. Our study showed that *ADRB2* gene polymorphisms were associated with the abundance of gut microbiota, suggesting that *ADRB2* gene polymorphisms may be a target for the prevention of gut microbiota-related diseases.

While some other experiments indicated that rs1042711 was associated with asthma susceptibility in Han Chinese children and that its T allele carriers had an increased risk of COPD [43,44], rs12654778 was also associated with COPD [45]. The gut microbiota in COPD patients is characterized by the presence of representatives of the Proteobacteria, such as *Citrobacter*, *Eggerthella*, *Pseudomonas*, *Anaerococcus*, and *Proteus* [46]. However, no association of rs1042711 and rs12654778 with these bacteria was found in our experiments, which may be related to the sample size, as well as the ethnicity of the participants.

To date, several studies have identified associations between the human gut microbiota and certain gene polymorphisms in the host [47,48,49], and because the establishment of the gut microbiota is a multifactorial process influenced by host genetics, diet, and physical activity, most of these studies have included different dietary interventions to explore the impact of host genetics on the relationship with the gut microbiota. In the present study, dietary interventions were not taken into account because the initial aim was to investigate whether *ADRB2* gene polymorphisms were associated with the human gut microbiota. In the future, these factors could be taken into account one by one, sample sizes and regions could be expanded, and additional genes could be screened to elucidate the mechanisms of gut microbiota–host interactions.

## 5 Conclusion

Our findings indicated that the relative abundance, diversity, and PICRUSt function prediction of gut microbiota were significantly different between males and females and that *ADRB2* gene polymorphisms were associated with the abundance of gut microbiota in Chinese Han adolescents. These findings provided new insights into the potential basis of *ADRB2* and provided evidence for a role of genetic factors. However, the human body is a complex system, there are many types of intestinal flora, and the relationships between different bacteria are complicated. Genetics experiments, including the present study, are affected by geographical region and sample size. In the future, we will aim to increase the sample size, expand the geographical region, and screen more genes to elucidate the mechanisms underlying the gut microbiota–host interaction.

## Supplementary Material

Supplementary Table
