# Supplementary material for: Association of ADRB2 gene polymorphisms and intestinal microbiota in Chinese Han adolescents
Source: Open Life Sci. 2023 Aug 1;18(1):20220646. doi: 10.1515/biol-2022-0646 (PMC10404897; doi:10.1515/biol-2022-0646)
Supplement: Supplementary Table [file biol-2022-0646-sm.pdf]

Supplementary material

Table S1: Association between *ADRB2* polymorphisms and total gut microbiota

SNP	Group	Phenotype	*N*	Effector allele	TEST	*β*	*P* value
rs12654778	total	c_Bacilli	91	A	ADD	−7,311	0.038
rs1042711	total	c_Erysipelotrichi	91	C	ADD	8,711	0.000
rs1801704	total	c_Erysipelotrichi	91	C	ADD	8,711	0.000
rs11959427	total	c_Erysipelotrichi	91	C	ADD	6,412	0.003
rs1042719	total	c_Erysipelotrichi	91	C	ADD	−2,581	0.019
rs12654778	total	o_Lactobacillales	91	A	ADD	−6,137	0.035
rs1042711	total	o_Erysipelotrichales	91	C	ADD	8,711	0.000
rs1801704	total	o_Erysipelotrichales	91	C	ADD	8,711	0.000
rs11959427	total	o_Erysipelotrichales	91	C	ADD	6,412	0.003
rs1042719	total	o_Erysipelotrichales	91	C	ADD	−2,581	0.019
rs1042711	total	o_Bifidobacteriales	91	C	ADD	5,966	0.030
rs1801704	total	o_Bifidobacteriales	91	C	ADD	5,966	0.030
rs2053044	total	f_Ruminococcaceae	91	A	ADD	3.32×10^4	0.030

Table S2: Association between *ADRB2* polymorphisms and male gut microbiota

SNP	Group	Phenotype	*N*	Effector allele	TEST	*β*	*P* value
rs1042711	male	p_Actinobacteria	41	C	ADD	1.48×10^4	0.048
rs1801704	male	p_Actinobacteria	41	C	ADD	1.48×10^4	0.048
rs2053044	male	c_Bacilli	41	A	ADD	1.01×10^4	0.015
rs1042711	male	c_Coriobacteriia	41	C	ADD	3,641	0.028
rs1801704	male	c_Coriobacteriia	41	C	ADD	3,641	0.035
rs2053044	male	o_Lactobacillales	41	A	ADD	1.02×10^4	0.007
rs1042711	male	o_Bifidobacteriale	41	C	ADD	1.23×10^4	0.035
rs1801704	male	o_Bifidobacteriale	41	C	ADD	1.23×10^4	0.035
rs2053044	male	f_Lachnospiraceae	41	A	ADD	4.46×10^4	0.037
rs2053044	male	f_Porphyromonadaceae	41	A	ADD	1.02×10^4	0.018
rs2053044	male	g_*Dialister*	41	A	ADD	-1.52×10^4	0.016
rs11959427	male	g_*Dialister*	41	C	ADD	-2.28×10^4	0.037

Table S3: Association between *ADRB2* polymorphisms and female gut microbiota

SNP	Group	Phenotype	*N*	Effector allele	TEST	*β*	*P* value
rs2053044	female	p_Firmicutes	50	A	ADD	-7.41×10^4	0.041
rs2053044	female	c_Clostridia	50	A	ADD	-7.30×10^4	0.043
rs1042711	female	c_Erysipelotrichi	50	C	ADD	1.45×10^4	0.000
rs1801704	female	c_Erysipelotrichi	50	C	ADD	1.45×10^4	0.000
rs11959427	female	c_Erysipelotrichi	50	C	ADD	9,658	0.009
rs1042719	female	c_Erysipelotrichi	50	C	ADD	3,569	0.043
rs2053044	female	o_Clostridiales	50	A	ADD	-7.30×10^4	0.043
rs1042713	female	o_Lactobacillales	50	G	ADD	8784	0.035
rs1042711	female	o_Erysipelotrichales	50	C	ADD	1.45×10^4	0.000
rs1801704	female	o_Erysipelotrichales	50	C	ADD	1.45×10^4	0.000
rs11959427	female	o_Erysipelotrichales	50	C	ADD	9,658	0.009
rs1042719	female	o_Erysipelotrichales	50	C	ADD	3,569	0.043
rs1042713	female	o_Bifidobacteriales	50	G	ADD	1,998	0.000
rs1042717	female	o_Bifidobacteriales	50	A	ADD	1,772	0.001
rs1042718	female	o_Bifidobacteriales	50	A	ADD	1,624	0.003
rs2053044	female	o_Bifidobacteriales	50	A	ADD	−1,631	0.014
rs1042719	female	o_Bifidobacteriales	50	C	ADD	−1,246	0.028
rs12654778	female	f_Bacteroidaceae	50	A	ADD	-6.24×10^4	0.045
rs2053044	female	f_Ruminococcaceae	50	A	ADD	-4.66×10^4	0.035
rs1042719	female	f_Ruminococcaceae	50	C	ADD	-3.85×10^4	0.041
rs1042717	female	f_Veillonellaceae	50	A	ADD	-2.44×10^4	0.041
rs12654778	female	g_*Bacteroides*	50	A	ADD	-6.24×10^4	0.045
rs2053044	female	g_*Faecalibacterium*	50	A	ADD	-2.85×10^4	0.029
